# Minimally Invasive Treatment of Spinal Metastases: Techniques

**DOI:** 10.1155/2011/494381

**Published:** 2011-06-28

**Authors:** Peter S. Rose, Michelle J. Clarke, Mark B. Dekutoski

**Affiliations:** ^1^Department of Orthopedic Surgery, Mayo Clinic, Rochester, MN 55905, USA; ^2^Department of Neurosurgery, Mayo Clinic, Rochester, MN 55905, USA

## Abstract

With improved treatments and increasingly life expectancy, the burden of metastatic disease in the spine is expected to rise. The role of conventional surgery for spinal metastases is well established but often involves procedures of large magnitude. We describe minimally invasive techniques for spinal stabilization and decompression in patients with symptomatic metastatic disease of the spine.

## 1. Introduction

Metastatic disease to the spine is an increasingly common clinical condition given improved cancer care and overall mortality trends. The majority of patients who die of cancer have vertebral metastases at autopsy [1, 2]. An estimated 20,000 patients per year in the United States have symptomatic epidural spinal cord compression and a much greater number have symptomatic vertebral metastases [3, 4]. The clinical burden in other areas of the world is likely similar. 

Surgical management is entertained in patients who have spinal instability and/or compressive neurologic deficits [5]. Although there are rare indications for formal en bloc resection of metastatic tumors, the vast majority of patients with metastatic disease in the spine are treated with palliative intent. These patients are often weakened as a consequence of their primary disease process as well as its attendant treatments (e.g., chemotherapy and radiotherapy). 

Open surgical treatment has a well-established clinical record in the treatment of patients with metastatic disease to the spine [6, 7]. However, advancements in surgical technique, instrumentation, and imaging modalities have led to the development of minimally invasive surgical techniques (MIS) in the treatment of metastatic disease to the spine. These procedures seek to decrease the physiologic insult, recovery time, and morbidity of more traditional open spine procedures. As well, they often allow more rapid initiation of other treatments (chemotherapy, radiotherapy) postoperatively than would be permitted by traditional approaches alone. MIS surgery of the spine seeks to achieve equivalent or superior outcomes to those of traditional open spine surgery with the use of these techniques. It is important to recognize, however, that the safety and efficacy of these techniques are currently under investigation and depend on surgeon experience and patient selection. This paper will review techniques commonly employed in minimally invasive spine surgery for metastatic disease. Percutaneous augmentation techniques (vertebroplasty/kyphoplasty) may be used in concert with these procedures but are not the focus of this paper.

## 2. Indications/Contraindications

Patients are considered for MIS treatment of spinal metastases if they require stabilization with or without tumor decompression. Relative contraindications to these techniques include circumferential tumors (in which adequate decompression may be difficult to achieve through an MIS approach alone). As well, highly vascular tumors (e.g., renal cell carcinoma) which require decompression with a high risk of resulting epidural hematoma may best be approached through open procedures in which proper hemostasis can be assured. Finally, it must be recognized that currently available MIS instrumentation is not as strong as the strongest traditional open instrumentation. Thus, patients who lack anterior column support should probably not undergo stand-alone MIS posterior instrumentation without concordant techniques to increase anterior column support (e.g., concurrent vertebro/kyphoplasty, surgical instillation of methylmethacrylate, or cage insertion). The recent development of cobalt chrome rods for use in MIS systems improves the biomechanical profile of these instrumentation sets but does not remove these concerns.

## 3. Surgical Techniques

Common surgical techniques used in minimally invasive surgery for spinal metastases include posterior pedicle screw-based stabilization applied percutaneously, limited open decompressive procedures, percutaneous extracavitary/costotransversectomy type approaches accessed through a paraspinal muscle splitting mechanism, and direct lateral approaches. These techniques may be used in combination and may be further coupled with vertebro/kyphoplasty procedures to maximize the repertoire of disease which may be addressed in this manner.

Excepting rare and unique circumstances, we do not recommend decompression without instrumentation in the treatment of metastatic disease. The presence of the tumor itself is generally a destabilizing influence on the spine, and the bony removal necessary for decompression leads to further destabilization. Even if the assessment at the time of surgery is that appropriate remaining bone stock is present, with progression of disease, there is a high likelihood of spinal instability developing. Thus, posterior instrumentation is a standard part of MIS spine tumor treatment in our hands.

Spinal stabilization without formal fusion is usually the goal of these procedures. We do not pursue an extensive decortication or bone grafting in the treatment of metastatic disease. Bony fusion is unlikely to ever be achieved in the hostile environment containing a tumor which is treated by adjuvant chemo- and/or radiotherapy. The decortication used to promote bony fusion disrupts natural anatomic barriers to further tumor infiltration and spread, increases the scope of the procedure beyond its oncologic goals, and removes/weakens further stabilizing bone stock. If cavities remain which require filling for structural support, we prefer to achieve this with the use of methylmethacrylate and/or further instrumentation. Note that the United States Food and Drug Administration considers spinal instrumentation without fusion in this manner to be an “off-label” use of these devices. The authors employ this technique for the clinical reasons outlined above and inform their patients of this discrepancy between how these devices are approved and how they are used in these unique clinical situations.

### 3.1. Technique: MIS Posterior Pedicle Screw Instrumentation

**Figure 1:**
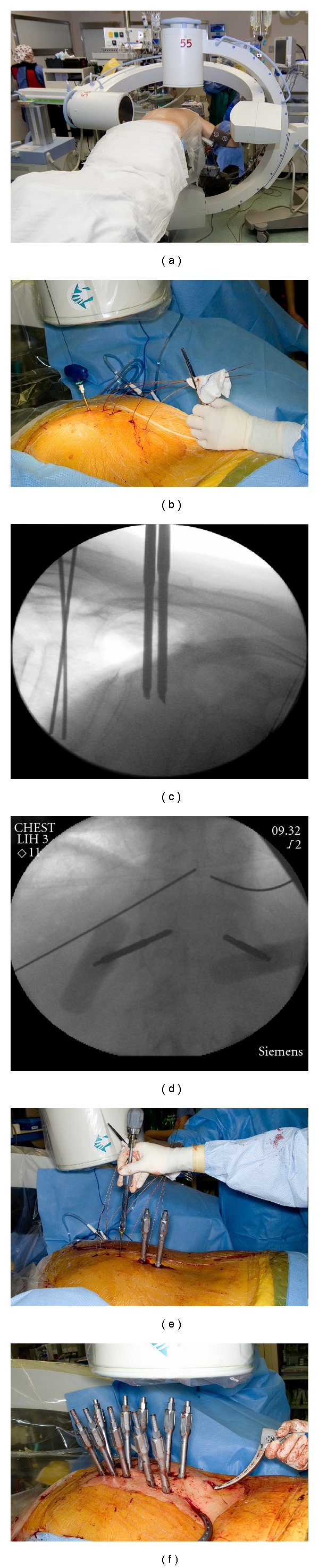
Percutaneous pedicle screw instrumentation. (a) Dual fluoroscopy setup; (b) percutaneous cannulation of pedicles; (c, d) lateral and anteroposterior imaging of pedicle cannulation; (e) passage of pedicle screws; (f) percutaneous passage of rod.

Percutaneous pedicle screw instrumentation is used in nearly all MIS metastatic tumor surgeries by our group (Figure 1). Patients are positioned prone upon a radiolucent operating table. Imaging is usually achieved through the use of biplanar fluoroscopy. Alternatively, an intraoperative CT scanner and stereotactic navigation with appropriate reference landmarks may be used at the surgeon's discretion. We prefer to drape in two fluoroscopy units to allow efficient AP and lateral imaging to be obtained.

At the discretion of the operating surgeon, instrumentation may be placed through a series of paraspinal stab incisions, two parallel longitudinal paraspinal incisions, or a single mid-line incision. If a single mid-line incision is used, dissection is generally taken down to the level of the dorsal interfascial plane, and instrumentation is then placed through small individual fascial incisions.

Using biplanar fluoroscopy, pedicles are cannulated using a cannulated awl. Guidewires are then placed through each cannula. Cannulated taps are used to establish the pedicle screw trajectory, and pedicle screws are then placed over these guidewires. The guidewire is removed once the screw is inserted. Screwdrivers or reduction towers may remain in place as a mechanism to allow manipulation of the vertebral body (to correct deformity) and provide for locking screws to be placed once the stabilizing posterior rods are in place. 

Once fixation points are all placed, rods are passed percutaneously through the screw heads and locked in place. A separate paraspinal stab incision is generally used to allow entry of the rods proximal to distal. In the lower lumbar spine, the lordotic orientation of L4-S1 sometimes makes it easier to pass rods from caudal to cranial. Once the rods are locked in place, the instruments used to place the screws are removed, the wound is irrigated and then closed in the standard fashion. 

**Figure 2:**
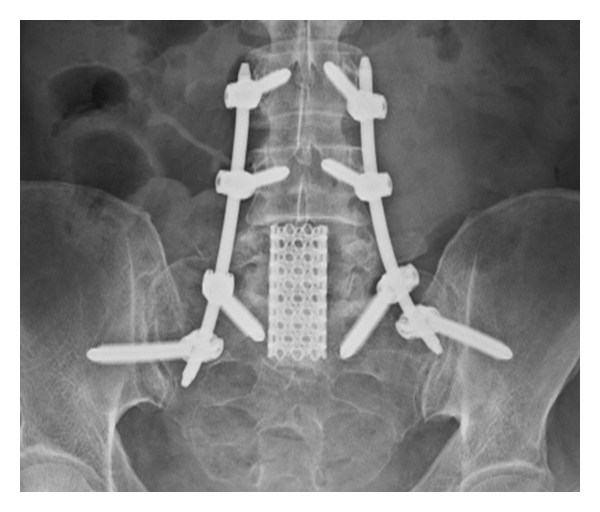
Use of percutaneous posterior instrumentation to stabilize open L5 corpectomy.

As patients with metastatic disease often have involvement at levels adjacent to the primary pathology, it is our preference to err on the side of a greater number of fixation points to distribute any stresses over a greater area, decreasing the risk of catastrophic failure. In practice, we generally instrument with bipedicular fixation two to three levels above and two to three levels below the site of disease in this manner. Instrumentation (or spannage) of six to ten levels is commonly achieved. Percutaneous instrumentation is also used to provide supplemental fixation for patients who have undergone limited open anterior procedures (Figure 2).

### 3.2. Combined Miniopen Decompression

In conjunction with the percutaneous instrumentation described above, miniopen decompression may be used to remove dorsal or dorsal-lateral areas of tumor compression. As well, in cases below the conus medullaris, anterolateral decompression may be achieved through this route as well with gentle manipulation of the thecal sac.

**Figure 3:**
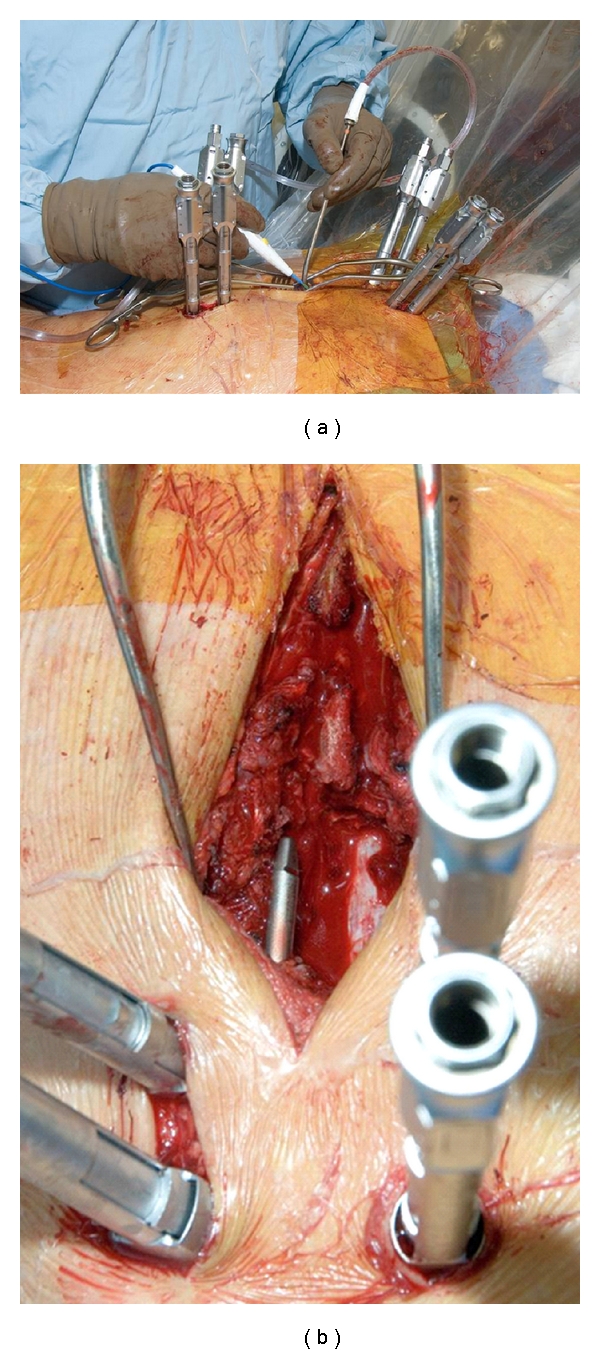
Miniopen decompression combined with percutaneous instrumentation. (a) Limited open decompression; (b) view of decompressed dural tube during rod passage.

Patients undergoing a miniopen posterior decompression have a short segment mid-line incision performed over the area of offending pathology. Dissection is then taken down to allow for limited laminectomy and tumor removal (Figure 3). Any dorsal tumor is easily removed via this approach. As well, the medial portion of the pedicle on either side may be removed and angled instruments can be used to achieve a decompression of dorsal-lateral, lateral, and anterolateral tumor compression.

This approach is very helpful when the surgeon suspects the tumor may be hypervascular. This miniopen approach allows proper exposure for hemostasis and a high degree of confidence that a postoperative hematoma is unlikely. Limited open approaches also provide the best access if unilateral rhizotomy are necessary to access offending tumor or to repair any dural tears which occur. No special imaging capabilities beyond basic fluoroscopy for level localization and percutaneous pedicle screw placement is necessary for this technique.

### 3.3. Percutaneous Extracavitary/Costotransversectomy Technique

**Figure 4:**
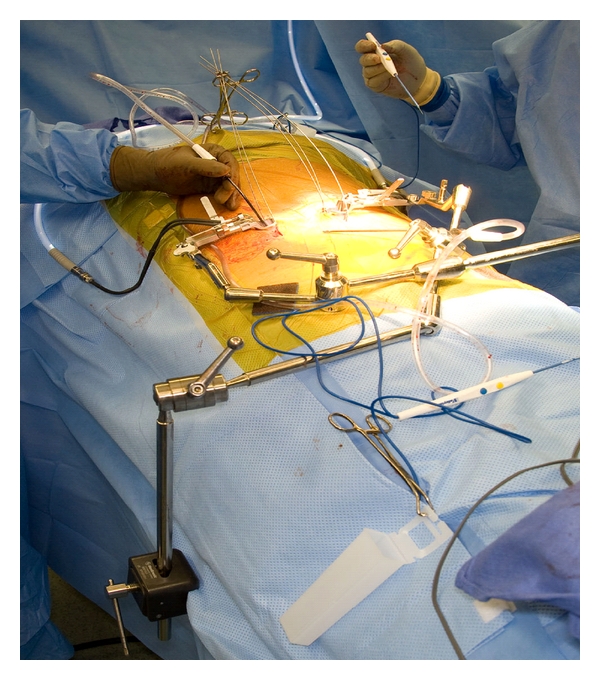
Setup for bilateral extracavitary approach showing dilating retractors inserted off midline rigidly attached to the operative table.

More extensive ventral and lateral tumor may be resected through the use of a percutaneous extracavitary/costotransversectomy approach. This utilizes the Wiltsie interval to access the lateral half of the dural tube as well as the ventral epidural space. This procedure requires more specialized instrumentation. Surgery relies upon the use of dilating percutaneous retractors which are docked over the surgical region of interest and held fixed by arms which attach to the operating table frame (Figure 4). These are usually used in conjunction with an operating microscope and bayoneted instruments to allow appropriate access.

Percutaneous extracavitary decompressions are coupled with posterior pedicle screw instrumentation placed percutaneously. The incision for the dilating retractor to allow the decompression is done 5 to 7 cm off the mid-line as dictated by local anatomy and the trajectory of the approach. Radiographic localization may be by fluoroscopy or via intraoperative navigation, depending on surgeon preference and facility capabilities. This technique allows more extensive decompression of ventral tumor masses than does a miniopen posterior approach alone. Access to the posterior vertebral body may be obtained for curettage of tumor or augmentation with cement. We have found that methylmethacrylate may be easily instilled into tumor cavities in the vertebral body accessed in this manner using a conventional PMMA cement gun with a small diameter nozzle typically used for implantation of shoulder and elbow arthroplasty components. When cement is injected in this manner, a cage, Steinmann pin, or small fragment screw is placed into the adjacent bone, and the cement is placed around it. This acts as an additional anchor to prevent migration of cement.

### 3.4. Direct Lateral Approach for Tumor Removal

Percutaneous systems now offer direct lateral access to the L1 through L4 vertebral bodies. Similar instruments may be used in conjunction with a minithoracotomy to access disease in the thoracic vertebral bodies. In much the same manner of an MIS extracavitary approach, a direct lateral approach may be used to access vertebral body disease. It is not our preference to resect epidural tumor extension through this approach as the visualization of the thecal sac is inferior to that achieved through an extracavitary approach. This approach does allow for resection of the anterior vertebral body and reconstruction with a cage or methylmethacrylate. Because of the overhanging iliac wing, the L5 vertebral body is not well accessed in this manner. 

## 4. Illustrative Case Example

**Figure 5:**
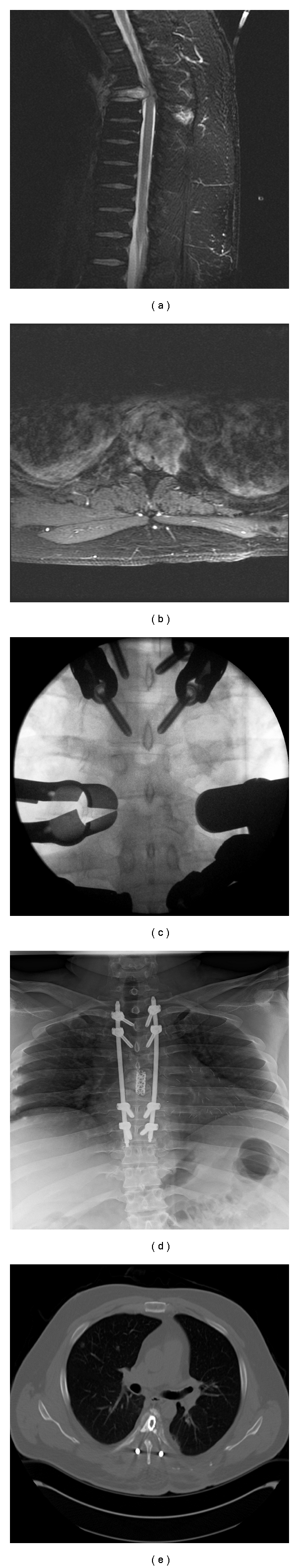
Bilateral extracavitary decompression for metastatic fibrosarcoma. (a) Sagittal T2-weighted, and (b) axial gadolinium enhanced MR images demonstrate pathologic fracture and metastatic epidural spinal cord compression at T5. (c) Fluoroscopy demonstrating docking of extracavitary retractors over the T5 costovertebral region. (d) Postoperate radiograph demonstrating T2–T8 fixation with cage + methylmethacrylate reconstruction of T5. (e) Postoperative CT demonstrates bilateral extracavitary decompression with cage/cement reconstruction.

Percutaneous fixation, bilateral extracavitary approach, and vertebral body reconstruction with cage and methylmethacrylate were employed in the successful treatment of a 37-year-old woman presenting with thoracic myelopathy from metastatic fibrosarcoma. Initial imaging studies demonstrate pathologic fracture of T5 with high-grade metastatic epidural spinal cord compression (Figures 5(a) and 5(b)). Patient underwent surgical treatment with percutaneous pedicle screw fixation spanning T2–T8 with bilateral extracavitary decompression at T5. Note that fluoroscopic imaging demonstrates docking of dilating retractors over the T5 costovertebral junction (Figure 5(c)). After tumor removal, a cage and methylmethacrylate were inserted to help reconstruct the weakened T5 body (Figure 5(d)). Post-operative CT demonstrates the resection achieved as well as cage/methylmethacrylate reconstruction. The patient recovered neurologic function post-operatively.

## 5. Discussion

Preliminary results of minimally invasive treatment of spinal metastases are encouraging [8]. Instrumentation failure is rare, and with proper adjuvant treatment, patients are unlikely to succumb to local mechanical or disease-related failure prior to demise. Most procedures can be performed with limited blood loss and modest operative times.

A benefit to the use of MIS approaches to metastatic spine tumors in our practice is a more rapid initiation of postoperative adjuvant therapies. For example, after a conventional open instrumented costotransversectomy procedure, we are hesitant to allow initiation of chemo- or radiotherapy until four to six weeks following the procedure to guard against wound dehiscence given the large approach, empty/dead space, and tissue devitalization which occurs with such procedures. In contrast, after an MIS extracavitary decompression and percutaneous instrumentation procedure, stereotactic radiotherapy may be initiated at our institution 10 to 14 days postoperatively. Although the percutaneous procedure may not allow a complete debulking of offending tumor from the vertebral body, the excellent local control (even of adverse histologies) achieved by stereotactic radiotherapy decreases the need for a gross total resection of tumor and may change the treatment paradigm of some patients with metastatic disease [9]. Our goal in the MIS treatment of these patients is to achieve a zone of decompression around the thecal sac of 2 to 4 mm followed by the rapid initiation of stereotactic radiotherapy to achieve local disease stasis. 

We emphasize that the adoption of these techniques by our surgical oncology group has been in a progressive fashion. There is certainly a learning curve associated with the implementation of MIS techniques in any setting. Given the altered anatomy and tissue conditions caused by tumor, radiotherapy, and so forth in the oncologic situation, this learning curve/case progression is highlighted. Our group began using MIS techniques for stabilization procedures alone and then expanded them to the use of stabilization coupled with miniopen decompressions and now pursue MIS extracavitary decompression of metastatic epidural spinal cord compression.

To date, our experience has been encouraged with low morbidity and favorable clinical and oncologic outcomes (Clarke et al., manuscript under review). Further experience will be necessary to refine the indications and techniques for MIS surgery applied to metastatic disease. Open surgical stabilization and decompression remain the gold standard to which MIS results must be compared. Direct comparative studies of open versus MIS treatments for metastatic disease in the spine are not currently available. Acknowledging these limitations, MIS techniques offer a valuable option for consideration in patients presenting with symptomatic metastatic disease of the spine.

## References

[1] Bach F, Larsen BH, Rohde K (1990). Metastatic spinal cord compression: occurrence, symptoms, clinical presentations, and prognosis in 398 patients with spinal cord compression. *Acta Neurochirurgica*.

[2] Perrin RG (1992). Metastatic tumors of the axial spine. *Current Opinion in Oncology*.

[3] Byrne TN, Benzel EC, Waxman SG, Byrne TN, Benzel EC, Waxman SG (2000). Epidural tumors. *Diseases of the Spine and Spinal Cord*.

[4] Posner JB (1995). *Spinal Metastases, in Neurologic Complications of Cancer*.

[5] Walker MP, Yaszemski MJ, Kim CW, Talac R, Currier BL (2003). Metastatic disease of the spine: evaluation and treatment. *Clinical Orthopaedics and Related Research*.

[6] Bilsky MH, Boland P, Lis E, Raizer J, Healey JH (2000). Single-stage posterolateral transpedicle approach for spondylectomy, epidural decompression, and circumferential fusion of spinal metastases. *Spine*.

[7] Patchell RA, Tibbs PA, Regine WF (2005). Direct decompressive surgical resection in the treatment of spinal cord compression caused by metastatic cancer: a randomised trial. *The Lancet*.

[8] Rose PS, Dekutoski MB, Clarke MJ Percutaneous instrumentation of pathologic vertebral fractures.

[9] Lovelock DM, Zelefsky M, Fuks Z (2008). High dose, single fraction image-guided intensity modulated radiotherapy for metastatic spinal. *International Journal of Radiation Oncology Biology Physics*.

